# Insecticidal and repellent activity of typical monoterpenes from plant essential oils against *Callosobruchus maculatus *(Fabr. 1775)

**DOI:** 10.1186/1753-6561-8-S4-P115

**Published:** 2014-10-01

**Authors:** Suelen L Reis, Anieli G Mantello, Èrica AG Rossete, Alexandre M Cardoso, Rene O Beleboni

**Affiliations:** 1 Unit of Biotechnology - Ribeirão Preto University (UNAERP), Ribeirão Preto, Brazil; 2 Federal Institute of Education, Science and Technology of São Paulo State (IFSP, Matão-SP), São Paulo, Brazil

## 

Synthetic insecticides and repellents have been broadly used worldwide for the control of crop pests and insects acting as vectors of human disease. Despite effectiveness, synthetic insecticides or repellents may cause serious damages to the environment and consequently to humans and animals. The growing biotechnological investigation for alternative insecticides and repellents, particularly of natural ones from plants, may conduce to higher safety and efficiency to the control of insects. *Callosobruchus maculatus *(Fabr. 1775) (Chrysomelidae) is a bruchid beetle that has been considered an important crop pest and also useful as model organism for development of new insecticides/repellents due several advantages such as a quick reproduction, sexual dimorphism and easy conditions of maintenance. As a crop pest, this insect, whose control by synthetic pesticides has been not straightforward, may represent an important threaten for bean (*Vigna unguiculata *L.) production in Brazil as well as other stored-grains cultivars in agriculture. Considering the need of new alternatives for *C. maculatus *control and the importance of monoterpenes in plant resistance against insects, the aim of this work was to evaluate the insecticidal and repellent activity of typical monoterpenes (geraniol, citral, ( ± ) citronellal, citronellol and eugenol) commonly found in plant essential oil against *C. maculatus*. To date, all of tested compounds presented a significant insecticidal and also repellent activity against *C. maculates *in a range of doses from 1 to 64 µL. The best tested compound as insecticide was the eugenol, while the best performance as repellent was accessed using eugenol. Thus, these data showed that evaluated monoterpenes presented significant insecticidal and repellent effects, which are of high biotechnological interest and useful towards the growth of agriculture worldwide.
